# Unraveling the Genetic and Environmental Risk Factors of Autism Spectrum Disorder Through a Case‐Control Study in Armenia

**DOI:** 10.1002/hsr2.70801

**Published:** 2025-05-04

**Authors:** Meri Mkhitaryan, Tamara Avetisyan, Hermine Yeritsyan, Hayk Harutyunyan, Konstantin Yenkoyan

**Affiliations:** ^1^ Neuroscience Laboratory COBRAIN Center, YSMU Yerevan Armenia; ^2^ COBRAIN Center YSMU Yerevan Armenia; ^3^ Muratsan University Hospital Complex, YSMU Yerevan Armenia

**Keywords:** autism spectrum disorder, case–control study, gene‐environment, genetics, pregnancy

## Abstract

**Background and Aims:**

Autism spectrum disorder (ASD) is influenced by genetic and environmental factors. This study investigates genetic mutations and prenatal factors associated with ASD, including their interplay, in a multicenter case‐control study.

**Methods:**

The study included 297 participants (149 ASD cases, 148 controls). Genetic data were analyzed using Multiplex Ligation‐dependent Probe Amplification (MLPA) for ASD‐associated chromosomal regions. Environmental data covered prenatal, perinatal, and neonatal factors. Logistic regression and stratified analyses were performed.

**Results:**

Significant associations were found between ASD and mutations in 15q11−15q13, 16p11, and 11q13 regions, particularly in *SHANK2* and *SHANK3*. Females with ASD showed higher mutation rates than males. Prenatal factors (e.g., MgB6 use, labor‐inducing drugs, maternal stress, pregnancy complications, shorter interpregnancy intervals) exacerbated ASD risk when combined with specific genetic variations. Duphaston use during pregnancy, alongside certain mutations, may influence ASD risk, potentially offering protective effects.

**Conclusion:**

This study highlights the importance of integrating genetic and environmental factors in ASD research. Findings underscore the need for tailored early interventions, prenatal care advice, and genetic counseling for at‐risk families. Future studies should validate these findings in larger, diverse populations and explore underlying mechanisms.

## 1 Introduction

Autism spectrum disorder (ASD) is a neurodevelopmental condition marked by social communication deficits and restricted behaviors. From 2012 to 2021, global prevalence in children is about 1% [1], likely underestimating cases in low‐ and middle‐income countries (LMICs). ASD affects diverse ethnic and socioeconomic groups [2]. Males are diagnosed four times more often than females, though this gap narrows with severity [3]. Data on prevalence in gender‐diverse communities is limited, and minority groups often receive later diagnoses [2, 4].

The exact cause of ASD is still unclear, but significant progress has been made in understanding its genetic and neurobiological aspects. Its etiology is multifactorial, involving both genetic and environmental factors [5, 6, 7, 8].

Twin and family studies suggest a strong genetic basis for ASD, with genetic contributions estimated between 50% and 95% in Europe and the US [9, 10]. Siblings of autistic individuals exhibit similar behaviors at rates of 3%−18% [11, 12]. Recent research has identified rare genetic variations, including inherited and de novo mutations, as well as copy number variations (CNVs) linked to autism [13, 14, 15]. The combined impact of numerous common genetic variations serves as a notable marker for ASD susceptibility [16, 17]. Over 200 genes associated with autism susceptibility have been identified, with key candidates like *CNTNAP2*, *CNTN4*, *SHANK3*, and *CHD2*. These genetic variants often converge on shared hereditary pathways.

Evidence shows that ASD is associated with over 20 pre‐, peri‐, and neonatal risk factors [18, 19, 20].

Despite a strong hereditary component, the phenotypic and genetic diversity of ASD indicates a complex origin. Research shows mixed results regarding environmental factors, with some studies highlighting genetic dominance and others suggesting equal contributions from both heritable and non‐heritable factors [21].

Prenatal and perinatal complications are believed to stem from interactions between genetic and environmental factors. An epidemiological study found that healthy siblings of autistic individuals experience fewer complications than their autistic counterparts, indicating reduced resilience to adverse prenatal experiences in those with ASD. Animal studies indicate that genetic abnormalities affecting synaptic function can heighten vulnerability to environmental influences [22] and link severe pathology in premature brains to disrupted synaptic development [23, 24]. Additionally, interactions between genetic variations in the melatonin pathway and oxidative stress [25], and enzyme deficits [26] may also play a role, along with prenatal and neonatal risk factors increasing the likelihood of hypoxia [27, 28].

Animal studies indicate gene‐environment (GxE) interactions in ASD risk. Ehninger et al. found that mice with *TSC2* gene haploinsufficiency displayed abnormal social behavior only with maternal immune activation [29] suggesting that TSC/mTOR signaling may amplify immune effects. Another study showed that prenatal maternal immune activation combined with a mutant DISC1 protein altered sociability in an animal model [30].

Research on GxE interactions in ASD is limited, with some studies [31, 32] highlighting the challenges of detection. Recent studies in other psychiatric disorders have shown limited positive results. However, these investigations are crucial for resolving inconsistencies in traditional research and informing prevention efforts.

Recent research indicates that epigenetic factors, such as DNA methylation, histone modifications, and microRNAs (miRNAs), play a significant role in autism predisposition [33, 34].

Research on GxE interactions in ASD is still in its early stages and remains limited for several reasons. While significant progress has been made in identifying genetic risk factors (e.g., de novo mutations, CNVs, and common variants) and environmental risk factors (e.g., prenatal exposure to air pollution, maternal immune activation, and advanced parental age), the interplay between these factors is poorly understood. Most studies to date have focused on investigating genetic and environmental contributions to ASD independently, rather than examining how they may interact to modulate risk.

One major challenge in studying GxE interactions is the complexity of both genetic and environmental factors. Genetic susceptibility to ASD is highly heterogeneous, involving hundreds of genes with small individual effects, while environmental exposures are often difficult to quantify and may vary widely across populations and time. Additionally, methodological limitations, such as small sample sizes, lack of longitudinal data, and insufficient statistical power to detect interactions, have hindered progress in this area.

Furthermore, there is a lack of consensus on the most appropriate statistical approaches and study designs for investigating GxE interactions in ASD. Many existing studies rely on candidate gene approaches, which may miss broader genetic contributions or fail to account for the multifactorial nature of ASD. Large‐scale, well‐powered studies that integrate genomic, epigenomic, and environmental data are needed to unravel the complex mechanisms underlying GxE interactions in ASD.

In Armenia and the broader region, research on ASD, particularly on GxE interactions, remains limited due to challenges such as restricted resources, insufficient infrastructure, and limited awareness of ASD as a public health priority. While global research on ASD has advanced significantly, populations in LMICs, including Armenia, remain underrepresented in these studies. This study is among the first to systematically explore both genetic and environmental risk factors for ASD in this region, providing critical insights into the interplay of these factors in a previously understudied population. By doing so, this study not only contributes to the global understanding of ASD but also lays the groundwork for future research and public health initiatives in Armenia and similar settings.

This study is the second phase of a larger project investigating ASD risk factors, focusing on genetic factors in Armenian children and their potential associations with key pre‐, peri‐, and neonatal risks outlined in our previous publication [20].

The objectives of the study are the following:

1. *Determine genetic risk factors*: Identify the genetic factors linked to an increased risk of ASD in children from Armenia.
2. *Explore gene‐environment interactions:* Examine how environmental factors during the pre‐, peri‐, and neonatal stages influence the effect of genetic risk factors on ASD development.

To our knowledge, this is the first study in Armenia to examine genetic and environmental risk factors associated with ASD and their potential interactions.

## 2 Methods

### 2.1 Study Design

A case‐control study design was employed to investigate the genetic and environmental risk factors associated with ASD and their interactions.

### 2.2 Study Population

The study included 297 participants: 149 children with ASD and 148 typically developing controls. The sample size was determined based on practical considerations and comparable studies [35, 36] rather than prior calculations. Recruitment occurred from 2021 to 2022, with age‐matched subjects aged 3−18 years. Children with ASD were recruited from the MY WAY Educational and Rehabilitation Center in Yerevan, with inclusion criteria requiring a DSM‐5 diagnosis and exclusion of other neurodevelopmental disorders. Control participants were randomly selected from the Muratsan Hospital Complex Policlinics during the same period. These children were registered as typically developing and had no documented history of neurodevelopmental or behavioral disorders. Their medical records were reviewed to confirm that they were attending the policlinic solely for routine check‐ups or minor conditions like the flu. This rigorous screening process ensured that the control group was free from neurodevelopmental or behavioral issues, providing a valid comparison group for the study.

### 2.3 Data Collection

#### 2.3.1 Genetic Data

##### 2.3.1.1 Genetic Testing Methodology

Genetic testing in this study was conducted to identify potential genetic variations associated with ASD. Blood samples were collected from participants using standard venipuncture techniques. Before collection, patient identity was confirmed, and an appropriate vein was selected to ensure a safe and efficient procedure. Blood was drawn into EDTA‐coated tubes to prevent clotting, as EDTA acts as an anticoagulant by chelating calcium ions, which are essential for the coagulation cascade. After collection, the tubes were gently inverted several times to ensure proper mixing of the blood with the anticoagulant, thereby maintaining sample integrity. Each sample was labeled with a unique identifier to ensure traceability and transported to the laboratory under controlled conditions to prevent degradation.

##### 2.3.1.2 DNA Extraction and Quality Control

Upon arrival at the laboratory, genomic DNA was extracted from the blood samples using a standardized protocol. The extracted DNA was quantified using spectrophotometric or fluorometric methods to ensure sufficient concentration and purity. Quality control measures, such as assessing the A260/A280 ratio (to confirm protein contamination levels) and gel electrophoresis (to check for DNA integrity), were performed to validate the suitability of the DNA for further analysis.

##### 2.3.1.3 Multiplex Ligation‐Dependent Probe Amplification (MLPA)

For genetic analysis, this study employed MLPA. MLPA was chosen for its ability to simultaneously detect CNVs in multiple genes or gene regions with high specificity and sensitivity, making it a cost‐effective and efficient method for identifying genomic imbalances associated with ASD. The target regions and genes were selected based on their well‐documented relevance to ASD and neurodevelopmental disorders, as identified through prior literature and genomic databases. This approach allowed us to focus on regions with known pathogenic potential while ensuring comprehensive coverage of clinically significant loci. This method is particularly useful for studying complex genetic disorders like ASD, where CNVs in specific genes or chromosomal regions are known to contribute to disease risk [37].

The SALSA MLPA Probemix P343 Autism‐1 and P396 *SHANK2* RUO assays were used in this study. These assays are designed to target chromosomal regions and genes that have been implicated in ASD, including [38]:

- *15q11‐q13*: This region contains several genes associated with neurodevelopmental disorders, including *UBE3A* (involved in ubiquitin‐mediated protein degradation), *GABRB3* (a subunit of the GABA‐A receptor, critical for inhibitory neurotransmission), and *CHRNA7* (a nicotinic acetylcholine receptor subunit linked to cognitive function).
- *16p11*: Recurrent CNVs in this region are strongly associated with ASD and other neurodevelopmental conditions.
- *22q13*: This region includes the *SHANK2* and *SHANK3* genes, which encode scaffolding proteins essential for synaptic function and plasticity. Mutations or CNVs in these genes are known to disrupt neural connectivity and are strongly linked to ASD.

#### 2.3.2 Environmental Data

The first part of our research investigated ASD risk factors, including sociodemographic details (age, sex, family history), prenatal factors (pregnancy complications, infections, stress, medication and supplement use, vitamin D levels), and maternal lifestyle factors (smoking, alcohol consumption, gestational weight gain). Peri‐ and neonatal factors such as gestational age, size, labor and delivery drug use, and mode of delivery were also examined, as detailed in our previous publication [20]. Data were collected through self‐reported questionnaires administered in face‐to‐face interviews with the child's parent.

### 2.4 Statistical Analysis

Statistical analyses were performed using SPSS 22. The statistical analysis was conducted in the following stages to investigate the genetic and environmental risk factors associated with ASD and explore potential GxE interactions. The environmental factors were selected from earlier research using logistic regression to identify pre‐, peri‐, and neonatal variables significantly correlated with ASD (*p *< 0.05) [20].

#### 2.4.1 Descriptive Statistics

Descriptive statistics were used to summarize the characteristics of the study population, including demographic, genetic, and environmental variables. Continuous variables (e.g., age, environmental exposure levels) were described using means, medians, and standard deviations, while categorical variables (e.g., sex, presence/absence of genetic mutations, exposure categories) were summarized using frequencies and percentages. The distribution of genetic mutations (e.g., *SHANK3* deletions, 16p11.2 duplications) and environmental exposures was compared between ASD cases and typically developing controls using appropriate statistical tests. For continuous variables, *t*‐tests were used, and for categorical variables, Chi‐square tests were applied. These analyses provided an overview of the study population and identified potential differences between cases and controls. A priori significance levels were set at *p* < 0.05.

#### 2.4.2 Stratification by Genetic Mutation Status

To explore potential GxE interactions, the study population was stratified based on the presence or absence of specific genetic mutations (e.g., *SHANK3* deletions, 16p11.2 duplications). Within each stratum, the association between environmental factors and ASD status was assessed using logistic regression or chi‐square tests, as appropriate. Stratum‐specific odds ratios (OR) and 95% confidence intervals (CI) were calculated to evaluate the effect of environmental exposures within each genetic subgroup. The strength and direction of these associations were compared across strata to identify potential interaction effects. For example, if the effect of an environmental factor on ASD risk was significantly stronger in individuals with a specific genetic mutation, this was interpreted as evidence of a GxE interaction.

All analyses were adjusted for potential confounders, including age, sex, and socioeconomic status, where applicable. To account for multiple comparisons, *p*‐values were adjusted using the Bonferroni correction, as appropriate.

## 3 Results

The analysis involved 297 participants in total, with 149 children diagnosed with ASD and 148 children without ASD. Basic demographic information (e.g., age, gender) and socioeconomic factors for both the case and control groups were previously described and statistically analyzed in the first part of this study [20]. To avoid redundancy, these details are not repeated here, but readers are encouraged to refer to the earlier publication for further information. Sample sizes for all analyses are provided in Tables S1 and S2, which include detailed results.

The descriptive analysis indicated notable disparities between the cases and controls across several genetic probes (Table S1).

### 3.1 Changes Across the 15q11−15q13 Chromosomal Regions

**Figure 1:**
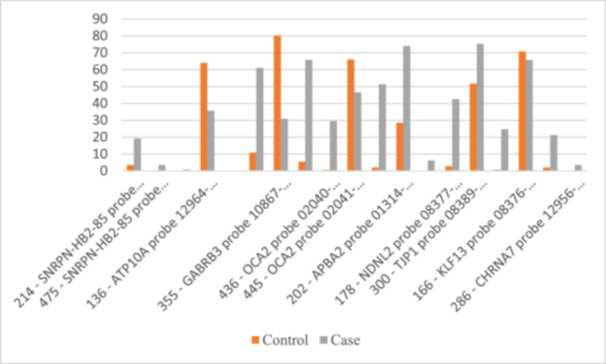
Distribution of statistically significant probes across the 15q11−15q13 chromosomal regions.

The analysis of the 15q11−15q13 chromosomal region indicate that several genetic probes showed significant differences in mutation frequencies between ASD cases and controls. Specifically, the probes for *SNRPN‐HB2‐85*, *ATP10A*, *GABRB3*, *OCA2*, *APBA2*, *NDNL2*, *TJP1*, *KLF13*, and *CHRNA7* exhibit increased frequencies of heterozygous and/or homozygous mutations in ASD cases, suggesting their potential involvement in ASD (Figure 1).

### 3.2 Changes Within the 16p11 Chromosomal Region

**Figure 2:**
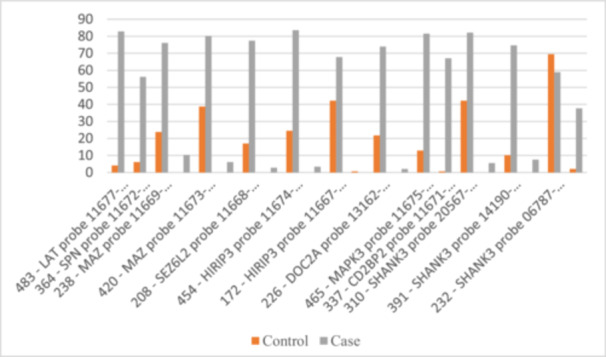
Distribution of statistically significant probes within the 16p11 chromosomal region.

The examination of the 16p11 chromosomal region highlighted several significant genetic variations associated with ASD. Notably, probes such as *LAT*, *SPN*, *MAZ*, *SEZ6L2*, and *SHANK3* showed highly significant differences between control and ASD cases, with increased mutation rates in the ASD group. This suggests a strong association between these genetic mutations and the occurrence of ASD (Figure 2).

### 3.3 Changes Within the 11q13 Chromosomal Region

**Figure 3:**
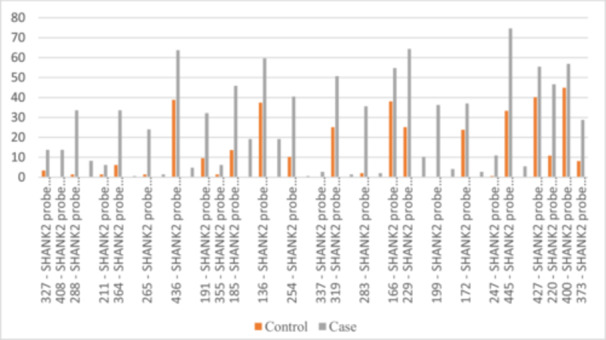
Distribution of statistically significant probes within the 11q13 chromosomal region.

Data from the 11q13 chromosomal region on the *SHANK2* gene revealed significant differences in mutation frequencies between ASD cases and controls. Many probes showed higher rates of heterozygous and homozygous mutations in ASD cases. Notable increases in heterozygous mutations were found in probes 327, 408, 211, 364, 265, 436, 191, 185, 136, 319, 283, 166, 229, 199, 172, 247, 445, 427, 220, and 400. Probes 288, 185, 136, 229, 199, 445, and 283 showed significant increases in both mutation types. This indicates a strong association between *SHANK2* variations and ASD (Figure 3).

These findings highlight the importance of specific genetic regions and mutations in understanding the genetic basis of ASD. The study identified several genetic probes with no mutations in the control group, including the *SNRPN‐HB2‐85* probe, multiple *UBE3A* probes, the *ATP10A* probe, several *GABRB3* probes, and *SCG5* probes. Additionally, *SHANK2* probes also showed no mutations in the control group.

### 3.4 Genetic Mutation Patterns by Gender

**Figure 4:**
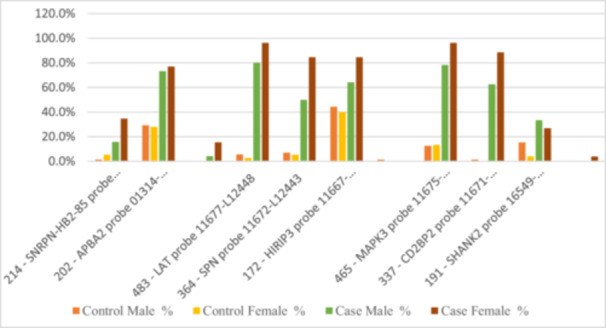
Distribution of statistically significant probes between case and control groups based on gender.

We analyzed genetic mutation distribution by gender across case and control groups for various probes (Figure 4, Table S2). In the 214 ‐ *SNRPN‐HB2*‐85 probe, 84.2% of males had no mutations compared to 65.4% of females, indicating higher mutation rates in females (34.6%). For the 202 ‐ *APBA2* probe, 22.5% of males had no mutations, while 7.7% of females had two. In the *LAT* probe, 20.0% of males had no mutations versus 3.8% of females, with females at 96.2%. The *SPN* probe showed 50.0% of males with no mutations compared to 15.4% of females. *HIRIP3* had 35.8% of males with no mutations versus 15.4% of females, and *MAPK3* had 21.7% of males with no mutations versus 3.8% of females. In the *CD2BP2* probe, 37.5% of males had no mutations compared to 11.5% of females. Overall, females exhibited higher mutation rates across multiple probes, suggesting significant gender‐specific patterns for future research.

### 3.5 Associations Between Genetic Mutations and Pre‐, Peri‐, and Neonatal Factors

Stratified analyses revealed significant associations between specific genetic mutations (probes) and various factors related to ASD. Below, we summarize the key findings, organized by the factors investigated (please see Table 1).

**Table 1:** Summary of stratified analysis among genetic mutations and various factors associated with ASD.

Probes	Male gender	MgB6 use during pregnancy	Labor‐inducing drug use	Self‐reported stress (first trimester)	Self‐reported stress (third trimester)	Self‐reported stress (whole pregnancy)	Pregnancy with complications	Interpregnancy interval < 1 year	Duphaston use during pregnancy
136 ‐ *ATP10A* probe	4.113 (2.334−7.248)	3.302 (1.553–7.018)	5.187 (2.653–10.139)	17 (1.993–145)	16.971 (3.972–72.521)	9.305 (2.133–40.596)	5.028 (2.608–9.693)	—	0.929 (0.864–0.998)
355 ‐ GABRB3 probe	5.529 (3.184–9.601)	1.278 (1.03–1.585)	3.649 (2.032–6.554)	6.325 (2.708–14.768)	7.245 (2.725–19.267)	—	3.819 (2.196–6.641)	—	—
445 ‐ *OCA2* probe	4.154 (2.33–7.407)	2.824 (1.361–5.859)	4.243 (2.199–8.187)	5.215 (2.228–12.205)	7.618 (2.607–22.262)	4.302 (1.435–12.899)	3.089 (1.793–5.322)	—	—
202 ‐ *APBA2* probe	3.875 (1.825–8.229)	3.941 (1.13–13.749)	4.179 (1.629–10.72)	7.209 (1.644–31.616)	—	—	3.364 (1.438–7.868)	—	—
300 ‐ *TJP1* probe	3.6 (1.882–6.888)	6.11 (2.088–17.879)	5.397 (2.499–11.657)	9.308 (2.775–31.222)	11.697 (2.727–50.171)	6.496 (1.486–28.406)	3.89 (1.961–7.715)	—	—
166 ‐ *KLF13* probe	—	—	3.627 (1.909–6.889)	13.929 (1.494–129.817)	10.957 (3.237–37.089)	6.364 (1.837–22.048)	3.274 (1.886–5.685)	—	—
238 ‐ MAZ probe	2.857 (1.25–6.529)	—	5.236 (1.741–15.753)	—	—	—	15.278 (2.191–106.519)	—	—
420 ‐ MAZ probe	4.414 (2.188–8.905)	—	3.92 (1.708–8.995)	29.667 (5.626–156.43)	8.33 (1.912–36.281)	4.174 (0.931–18.712)	4.622 (1.952–10.945)	—	—
208 ‐ SEZ6L2 probe	2.758 (1.093–6.96)	—	3.629 (1.17–11.256)	8.99 (2.681–30.148)	9.307 (2.511–34.499)	3.961 (0.502–31.239)	10.684 (1.548–73.737)	—	—
454 ‐ *HIRIP3* probe	2.771 (1.251–6.138)	—	5.889 (1.964–17.661)	9.877 (2.641–36.939)	11.17 (1.467–85.035)	5.625 (0.725–43.656)	4.913 (1.63–14.81)	—	—
172 ‐ *HIRIP3* probe	3.281 (1.65–6.525)	4.917 (1.622–14.907)	4.741 (2.039–11.027)	12.636 (2.895–55.145)	11.935 (2.725–52.274)	12.4 (1.607–95.667)	4.836 (2.012–11.628)	—	—
465 ‐ *MAPK3* probe	—	1.198 (1.102–1.302)	6.125 (1.352–27.74)	—	6.971 (1.95–24.922)	—	—	—	—
310 ‐ *SHANK3* probe	—	3.184 (1.255–8.079)	3.801 (1.721–8.397)	24.286 (2.527–233.444)	8.3 (1.277–53.949)	3.336 (0.948–11.736)	3.157 (1.603–6.219)	—	—
391 ‐ *SHANK3* probe	—	1.161 (1.077–1.253)	4.007 (0.864–18.585)	11.206 (3.547–35.402)	9.284 (2.674–32.24)	11.882 (3.176–44.456)	—	—	—
232 ‐ *SHANK3* probe	—	5.657 (2.284–14.01)	4.124 (2.168–7.846)	7.724 (2.925–20.391)	—	5.862 (1.7–20.21)	4.278 (2.298–7.964)	—	—
136 ‐ *SHANK2* probe	—	3.267 (1.188–8.983)	5.404 (2.137–13.665)	10.095 (2.323–43.878)	15.955 (2.104–120.974)	8.471 (1.094–65.604)	4.304 (1.81–10.236)	—	—
229 ‐ *SHANK2* probe	—	4.058 (1.157–14.224)	5.207 (1.884–14.39)	6.955 (1.576–30.69)	10.41 (1.356–79.917)	8.4 (2.047–34.464)	4.979 (1.643–15.084)	—	—
400 ‐ *SHANK2* probe	—	5.25 (1.705–16.168)	8.25 (2.992–22.748)	11.353 (3.669–35.126)	21.725 (4.864–97.04)	13.167 (2.9–59.787)	2.697 (1.384–5.258)	—	—
445 ‐ *SHANK2* probe	—	—	5.321 (2.098–13.495)	9.343 (2.145–40.695)	7.293 (1.661–32.022)	7.462 (1.294–43.011)	5.026 (1.917–13.177)	—	0.899 (0.831–0.972)
427 ‐ *SHANK2* probe	—	—	3.165 (1.399–7.159)	8.777 (2.817–27.344)	9.532 (2.64–34.412)	9.667 (2.083–44.865)	2.428 (1.249–4.722)	—	—
436 ‐ *SHANK2* probe	—	—	5.263 (2.171–12.761)	8.963 (2.361–34.032)	11.646 (3.113–43.567)	4.797 (1.056–21.793)	2.921 (1.474–5.79)	—	—
166 ‐ *SHANK2* probe	—	—	3.48 (1.497–8.092)	12.754 (3.6–45.178)	11.981 (2.619–54.81)	15.789 (1.968–126.669)	3.781 (1.7–8.409)	—	—
226 ‐ *DOC2A* probe	—	—	—	8.88 (2.79–28.267)	—	5.433 (0.695–42.496)	—	—	—
483 ‐ *LAT* probe	—	—	—	—	—	—	—	3.421 (1.148–10.194)	—

*Note:* 1. The table includes only the probes which showed statistically significant (*p* < 0.05) association with the chosen pre‐, peri‐, and neonatal variables. 2. Each cell contains the odds ratio (OR) and 95% confidence interval (CI) in the format: OR (lower–upper). 3. A dash (—) indicates that the probe was not associated with that specific factor. 4. The table is organized by probes and includes all relevant factors for each probe.

### 3.6 Male Gender and Genetic Mutations

Several genetic mutations showed significant associations with male gender: the *ATP10A* probe demonstrated a strong association, with males having 4.1 times higher odds of this mutation compared to females (OR = 4.113, 95% CI: 2.334–7.248). Similarly, the *GABRB3* probe was strongly associated with male gender (OR = 5.529, 95% CI: 3.184–9.601). Other probes, such as OCA2 (OR = 4.154, 95% CI: 2.33–7.407) and TJP1 (OR = 3.6, 95% CI: 1.882–6.888), also showed significant but slightly weaker associations.

### 3.7 MgB6 Use During Pregnancy and Genetic Mutations

The use of MgB6 during pregnancy was associated with specific genetic mutations: the *ATP10A* probe showed a significant association (OR = 3.302, 95% CI: 1.553–7.018), suggesting that MgB6 use during pregnancy is linked to ~3.3 times higher odds of this mutation. The *GABRB3* probe exhibited a weaker but still significant association (OR = 1.278, 95% CI: 1.03–1.585).

### 3.8 Labor‐Inducing Drugs Use and Genetic Mutations

Labor‐inducing drug use was strongly associated with several genetic mutations: the *ATP10A* probe showed a particularly strong association (OR = 5.187, 95% CI: 2.653–10.139), indicating that labor‐inducing drug use is linked to ~5.2 times higher odds of this mutation. Other probes, such as GABRB3 (OR = 3.649, 95% CI: 2.032–6.554) and *TJP1* (OR = 5.397, 95% CI: 2.499–11.657), also demonstrated significant associations.

### 3.9 Self‐Reported Stress During Pregnancy and Genetic Mutations

Self‐reported stress during pregnancy (first trimester, third trimester, or whole pregnancy) was significantly associated with multiple genetic mutations. The *ATP10A* probe showed a very strong association with first‐trimester stress (OR = 17, 95% CI: 1.993–145), although the wide CI suggests some uncertainty in the estimate. Third‐trimester stress was significantly associated with the *GABRB3* probe (OR = 7.245, 95% CI: 2.725–19.267) and the *OCA2* probe (OR = 7.618, 95% CI: 2.607–22.262). Whole‐pregnancy stress was also associated with several probes, including ATP10A (OR = 9.305, 95% CI: 2.133–40.596) and TJP1 (OR = 6.496, 95% CI: 1.486–28.406).

### 3.10 Pregnancy Complications and Genetic Mutations

Pregnancy complications were significantly associated with multiple genetic mutations: the *ATP10A* probe showed a strong association (OR = 5.028, 95% CI: 2.608–9.693), indicating that pregnancy complications are linked to ~5 times higher odds of this mutation. Other probes, such as GABRB3 (OR = 3.819, 95% CI: 2.196–6.641) and *TJP1* (OR = 3.89, 95% CI: 1.961–7.715), also demonstrated significant associations.

### 3.11 Interpregnancy Interval (IPI) Less Than a Year and Genetic Mutations

Only one probe, the *LAT* probe, showed a significant association with a short IPI. The LAT probe was associated with ~3.4 times higher odds of this mutation in cases where the IPI was less than 1 year (OR = 3.421, 95% CI: 1.148–10.194).

### 3.12 Duphaston Use and Genetic Mutations

Duphaston use was associated with a small but statistically significant reduction in the odds of the *ATP10A* mutation (OR = 0.929, 95% CI: 0.864–0.998). Also, Duphaston use was associated with a stronger reduction in the odds of the SHANK2 mutation (OR = 0.899, 95% CI: 0.831–0.972). Overall, Duphaston use during pregnancy appeared to have a protective effect against specific genetic mutations, particularly the ATP10A and SHANK2 probes. This suggests that Duphaston may play a role in reducing the risk of these mutations, though further research is needed to confirm these findings and explore the underlying mechanisms.

## 4 Discussion

This study provides strong evidence of genetic and environmental risk factors for ASD, highlighting its multifactorial nature through an analysis of genetic mutations and prenatal factors. Our findings confirm a significant association between genetic probes in the 15q11−q13, 16p11, and 11q13 regions and ASD, reinforcing the critical role of these loci in the genetic architecture of ASD. Probes targeting genes such as *SNRPN*, *ATP10A*, *GABRB3*, and *SHANK2* exhibited higher rates of mutations in ASD cases compared to controls, aligning with previous research that underscores the contribution of these genes to ASD etiology [39, 40, 41].

The 15q11−q13 region is a well‐established locus implicated in neurodevelopmental disorders, including ASD. In our study, probes for *SNRPN*, *ATP10A*, and *GABRB3* showed significant associations with ASD. *SNRPN* (Small Nuclear Ribonucleoprotein Polypeptide N) plays a crucial role in mRNA splicing and gene regulation. Dysregulation of *SNRPN* has been linked to Prader−Willi and Angelman syndromes, which share phenotypic overlaps with ASD, including intellectual disability and behavioral abnormalities. The observed mutations in *SNRPN* may disrupt synaptic function and neuronal communication, contributing to ASD phenotypes [42]. *ATP10A* (ATPase Phospholipid Transporting 10A) is involved in phospholipid transport and membrane integrity. Mutations in this gene may impair neuronal membrane stability and signaling, potentially affecting synaptic plasticity and connectivity. *GABRB3* (Gamma‐Aminobutyric Acid Type A Receptor Subunit Beta3) encodes a subunit of the GABA‐A receptor, which mediates inhibitory neurotransmission. Altered *GABRB3* expression or function has been associated with ASD and epilepsy, suggesting that disruptions in GABAergic signaling may underlie the excitatory‐inhibitory imbalance observed in ASD.

The 16p11.2 region is one of the most consistently replicated loci in ASD research [43]. In our study, probes for *SHANK3* and *SEZ6L2* within this region showed significant differences between ASD cases and controls. *SHANK3* (SH3 and Multiple Ankyrin Repeat Domains 3) is a key synaptic scaffolding protein that regulates the structural and functional integrity of excitatory synapses. Mutations in *SHANK3* have been strongly associated with ASD, intellectual disability, and Phelan−McDermid syndrome [44, 45]. The observed mutations in *SHANK3* likely disrupt synaptic organization and signaling, leading to impaired neuronal connectivity and social communication deficits characteristic of ASD. *SEZ6L2* (Seizure‐Related 6 Homolog‐Like 2) is involved in neuronal development and synaptic function. Although less studied than *SHANK3*, *SEZ6L2* has been implicated in neurodevelopmental disorders, and its dysregulation may contribute to the synaptic and behavioral abnormalities observed in ASD.

The 11q13 region, particularly the *SHANK2* gene, emerged as a significant locus in our study. *SHANK2* encodes a synaptic scaffolding protein critical for glutamatergic synapse formation and function. Increased *SHANK2* mutation rates highlight the role of synaptic genes in ASD pathology [46], with disruptions potentially leading to synaptic dysfunction, affecting learning, memory, and social behavior—core features of ASD.

The convergence of mutations in *SHANK3*, *SHANK2*, and *GABRB3* underscores the centrality of synaptic dysfunction in ASD. These genes are essential for synapse formation, maintenance, and plasticity, and their dysregulation may disrupt excitatory‐inhibitory signaling, contributing to ASD symptoms like social communication deficits, repetitive behaviors, and sensory sensitivities. Additionally, genes like *SNRPN* and *ATP10A* highlight the broader impact of transcriptional regulation and membrane integrity on neuronal function and ASD risk.

Our analysis revealed a notable gender‐specific pattern, with females diagnosed with ASD exhibiting higher mutation rates compared to males. This finding aligns with the “female protective effect” hypothesis, which posits that females may require a greater genetic or mutational burden to manifest ASD [47, 48]. The observed gender disparity in mutation rates supports the growing body of evidence suggesting that females are inherently more resilient to genetic risk factors for ASD [49]. Several mechanisms may contribute to this protective effect:

*Biological resilience*: Females may possess intrinsic biological factors, such as enhanced DNA repair mechanisms or greater neural plasticity, that mitigate the impact of genetic mutations. These factors could buffer against the development of ASD phenotypes, requiring a higher mutational load to overcome this resilience.

*Genetic differences*: The presence of a second X chromosome in females may provide a compensatory mechanism. X‐linked genes associated with neurodevelopment may be differentially expressed or regulated in females, offering protection against ASD‐related mutations. In contrast, males, with a single X chromosome, may be more vulnerable to the effects of such mutations.

*Hormonal influences*: Sex hormones, such as estrogen, have been shown to play a neuroprotective role by promoting synaptic plasticity and reducing neuroinflammation. Higher levels of estrogen in females may confer additional protection against the development of ASD, whereas males may lack this hormonal safeguard.

*Compensatory mechanisms*: Females may exhibit greater social and cognitive compensatory abilities, allowing them to mask or adapt to ASD‐related traits. This could result in a higher threshold for clinical diagnosis, with only those females carrying a significant mutational burden meeting diagnostic criteria.

The stratified analysis in our study revealed significant interactions between genetic mutations and prenatal factors, highlighting the complex interplay between genetic predispositions and environmental exposures in ASD risk. Some of the 95% CIs in our analyses were relatively wide, which may reflect the variability in the data or the modest sample size for certain subgroups. While this limits the precision of some estimates, the findings remain informative for generating hypotheses and guiding future research. Larger studies with increased statistical power are needed to refine these estimates and confirm the observed associations. The use of magnesium vitamin B6 (MgB6) and labor‐inducing drugs during pregnancy was associated with increased odds of ASD when combined with certain genetic mutations. These interactions suggest that environmental exposures may amplify genetic vulnerabilities, increasing ASD risk. Potential mechanisms include neurodevelopmental disruption, where prenatal exposures may interfere with neuronal migration, synaptogenesis, and myelination, particularly when combined with genetic mutations affecting synaptic function (e.g., *SHANK3*, *GABRB3*). Placental function may also be compromised, altering fetal development, with genetic mutations in placental genes exacerbating these effects. Maternal stress and immune activation triggered by labor‐inducing drugs could release pro‐inflammatory cytokines, impacting fetal brain development, especially in cases of impaired immune regulation. Additionally, exposures may increase oxidative stress, causing DNA damage and mitochondrial dysfunction, with mutations in antioxidant pathways further raising ASD susceptibility [50, 51, 52].

Our findings align with a growing body of literature emphasizing the role of GxE interactions in ASD risk [53]. For example, prenatal exposure to medications like valproic acid or SSRIs can interact with genetic vulnerabilities, increasing ASD risk. Maternal immune activation (e.g., from infection or stress) can disrupt fetal brain development, particularly when combined with genetic risk factors. Oxidative stress plays a key role in ASD, with mutations in antioxidant pathways (e.g., SOD1, GSTP1) heightening susceptibility to environmental insults.

Maternal stress during the first and third trimesters, as well as throughout pregnancy, significantly increases ASD risk, especially when combined with specific genetic mutations. This highlights the complex interplay between environmental stressors and genetic vulnerabilities in shaping neurodevelopmental outcomes [50]. Maternal stress contributes to ASD risk through multiple mechanisms, often interacting with genetic factors. First, stress activates the maternal HPA axis, elevating cortisol levels that cross the placenta and disrupt fetal brain development, particularly in regions like the amygdala and prefrontal cortex—effects worsened by mutations in stress response genes (e.g., NR3C1) [54]. Second, stress can induce epigenetic modifications (e.g., DNA methylation) in genes critical for synaptic plasticity (BDNF) or neuroinflammation (IL6), with mutations in epigenetic regulators (MECP2) further amplifying ASD risk by impairing neuronal connectivity [55, 56]. Third, stress triggers neuroinflammatory responses, releasing cytokines (IL‐6, TNF‐α) that disrupt neurodevelopment, exacerbated by immune‐related genetic mutations (*C4A*, *HLA‐DRB1*) [57]. Fourth, stress elevates oxidative stress, causing DNA damage and mitochondrial dysfunction, with mutations in antioxidant pathways (*SOD1*, *GSTP1*) increasing susceptibility to synaptic and neuronal deficits [51]. Finally, stress may impair placental function, reducing nutrient and oxygen delivery, while mutations in placental transporters (*SLC2A1*) or stress responses further heighten neurodevelopmental risks [58].

Pregnancy complications, such as pre‐eclampsia, gestational diabetes, and placental insufficiency, significantly increase ASD risk, especially when combined with specific genetic mutations. Pregnancy complications disrupt fetal neurodevelopment through multiple pathways. Impaired placental function can reduce nutrient and oxygen delivery, causing intrauterine growth restriction (IUGR) or hypoxia, with genetic mutations in stress resilience or nutrient transport worsening these effects. Maternal inflammatory conditions (e.g., pre‐eclampsia, infections) release pro‐inflammatory cytokines that impair fetal brain development, particularly when combined with immune‐regulating genetic mutations. Complications also increase oxidative stress, damaging neurons and synaptic function—an effect amplified by mutations in antioxidant pathways. Hormonal imbalances from disorders like gestational diabetes or thyroid dysfunction may interact with genetic vulnerabilities in neuroendocrine pathways. Collectively, these complications create a hostile intrauterine environment that exacerbates genetic risks in stress response, synaptic function, and metabolic regulation, magnifying neurodevelopmental disruptions linked to ASD.

A shorter IPI is associated with increased ASD risk, particularly in cases involving mutations linked to the *LAT* probe. A short (PI can adversely affect maternal and fetal health through multiple pathways. Rapid successive pregnancies may deplete maternal folate, iron, and other nutrients essential for neurodevelopment, exacerbating risks for fetuses with mutations impairing nutrient metabolism or stress resilience. Shorter IPIs also elevate maternal stress, increasing cortisol levels via HPA axis activation and disrupting fetal brain development, particularly in those with genetic vulnerabilities in stress response pathways. Inadequate recovery time between pregnancies may compromise maternal health, increasing risks of anemia or hypertension and creating a suboptimal intrauterine environment. Additionally, short IPIs can impair placental development, reducing oxygen and nutrient delivery and potentially leading to IUGR or hypoxia—effects amplified in fetuses with mutations affecting stress resilience or nutrient transport. The *LAT* probe, involved in neuronal signaling and immune regulation, may mediate these effects, with mutations in *LAT* or related pathways potentially worsening neurodevelopmental disruptions from nutritional deficits, stress, or placental dysfunction, thereby increasing ASD risk [59, 60].

The analysis revealed that Duphaston (a progestin) use during pregnancy was associated with reduced odds of ASD, particularly when combined with certain genetic mutations. These findings suggest a potential protective effect of Duphaston, contrasting with prior studies associating progestin exposure with increased ASD risk. The observed benefit may arise through multiple mechanisms: stabilization of progesterone signaling (supporting placental function, reducing uterine contractions, and promoting neurodevelopment), anti‐inflammatory effects that mitigate maternal‐fetal inflammation, and enhancement of antioxidant pathways to reduce oxidative stress—particularly beneficial for fetuses with stress‐resilience mutations. Additionally, the protective effect may be amplified in individuals with genetic mutations affecting progesterone receptors or related pathways, demonstrating a critical GxE interaction.

Our findings contrast with those of Li et al., who reported an increased risk of ASD associated with prenatal progestin exposure. This discrepancy may be due to differences in the type of progestin used, dosage, timing of exposure, or population genetics. For instance, Duphaston's selective action on progesterone receptors, unlike other synthetic progestins, may avoid neurodevelopmental disruptions observed in other studies [61].

Overall, this study highlights the complex interplay between genetic mutations and prenatal factors in ASD development. The significant associations underscore the need to consider both genetic and environmental factors in ASD research, paving the way for future investigations.

Notably, this is the first study of its kind in Armenia, examining various genetic and environmental risk factors and their interactions.

### 4.1 Strengths and Limitations

#### 4.1.1 Strengths

This study offers several key advantages through its rigorous methodology. The case‐control design enables robust comparisons between ASD cases and typically developing controls, facilitating simultaneous examination of genetic and environmental risk factors. Comprehensive data collection integrates both biological and environmental variables, providing a holistic perspective on ASD etiology. Technically, the application of advanced MLPA techniques ensured sensitive detection of genetic abnormalities, while structured questionnaires with response verification enhanced environmental data reliability. Methodological rigor in study design and execution promoted high‐quality, reproducible results. Furthermore, the inclusion of diverse prenatal, perinatal, and neonatal factors increases the real‐world applicability of findings, making them particularly valuable for clinical and public health implementation.

#### 4.1.2 Limitations

This study has several limitations to consider. First, while structured questionnaires with response clarification were used, the reliance on self‐reported prenatal/environmental data may introduce recall bias, suggesting future studies should incorporate medical records for validation. The sample size, determined by practical constraints rather than power calculations, may limit statistical power and generalizability. Although MLPA effectively detected CNVs and exon‐level variants, its inability to identify point mutations, small indels, or variants outside probe regions represents a technical constraint. The analysis didn't stratify participants by ASD symptom severity, which could mask important heterogeneity, nor did it account for potential age‐related variability across the 3–18‐year range despite statistical controls. Finally, wide CIs in some analyses reflect sample size limitations, particularly in subgroups, though the findings still offer valuable preliminary insights. These limitations highlight opportunities to strengthen future research through larger, more phenotypically detailed studies incorporating complementary genetic techniques.

## 5 Conclusion

This study delves into the intricate interplay between genetic mutations and prenatal factors in the development of ASD. We confirm significant associations between specific genetic probes in the 15q11–15q13, 16p11, and 11q13 regions, underscoring the critical roles of *SHANK2* and *SHANK3* in ASD pathogenesis. Notably, gender‐specific patterns suggest that females may require a higher mutation burden or exhibit distinct mutation profiles compared to males, highlighting the importance of considering sex differences in ASD research.

The interactions between genetic mutations and prenatal factors– such as MgB6 use, labor‐inducing drugs, maternal stress, and pregnancy complications–reveal the multifactorial nature of ASD. Our findings demonstrate that these environmental exposures significantly amplify ASD risk when combined with specific genetic vulnerabilities. Furthermore, a shorter interpregnancy interval and the use of Duphaston in conjunction with certain mutations may influence ASD risk, with Duphaston potentially offering protective effects that warrant further exploration.

In summary, these results emphasize the necessity of a comprehensive, integrated approach to ASD research, incorporating both genetic and environmental factors. Future studies should prioritize randomized controlled trials to validate these findings and elucidate the underlying biological mechanisms. Clinically, healthcare providers should consider the impact of genetic predispositions and prenatal exposures to enhance early identification and intervention strategies for at‐risk populations. By advancing our understanding of these complex interactions, we can move closer to personalized prevention and treatment approaches for ASD.

### 5.1 Future Directions

The findings of this study have several important implications for public health interventions and policy, particularly in the context of Armenia and other LMICs with limited resources for ASD research and support. First, the identification of specific genetic and environmental risk factors for ASD highlights the need for targeted public health initiatives aimed at reducing modifiable environmental exposures, such as maternal health interventions during pregnancy and improved access to prenatal care. For example, raising awareness about the potential risks associated with certain environmental factors and promoting healthier prenatal practices could help mitigate some of the preventable contributors to ASD.

Second, the scarcity of research on ASD in Armenia underscores the importance of establishing national registries and surveillance systems to systematically collect data on ASD prevalence, risk factors, and outcomes. Such initiatives would not only improve our understanding of ASD in the region but also inform evidence‐based policies and resource allocation. Additionally, integrating genetic screening and counseling into public health programs could help identify at‐risk families and provide them with early intervention and support.

Finally, this study lays the groundwork for future research in Armenia and similar settings. Larger, population‐based studies with diverse cohorts are needed to validate and expand upon these findings. Incorporating advanced genomic technologies, such as whole‐exome or whole‐genome sequencing, could provide a more comprehensive understanding of the genetic architecture of ASD in underrepresented populations. Collaborative efforts between researchers, policymakers, and healthcare providers will be essential to translate these findings into actionable strategies that improve the lives of individuals with ASD and their families.

## Author Contributions

**Meri Mkhitaryan:** conceptualization, data curation, formal analysis, investigation, methodology, writing – original draft, writing – review and editing. **Tamara Avetisyan:** data curation, formal analysis, investigation, methodology. **Hermine Yeritsyan:** data curation, formal analysis, investigation, methodology. **Hayk Harutyunyan:** data curation, formal analysis, investigation, methodology, validation, writing – review and editing. **Konstantin Yenkoyan:** conceptualization, formal analysis, funding acquisition, methodology, project administration, supervision, writing – original draft, writing – review and editing.

## Ethics Statement

The study protocol received approval from the Ethics Committee of Yerevan State Medical University (N 8–2/20; 27.11.2020), adhering to the principles of the Declaration of Helsinki.

## Consent

Written informed consent was obtained from all parents of the participants before data collection began.

## Conflicts of Interest

The authors declare no conflicts of interest.

## Transparency Statement

The lead author, Konstantin Yenkoyan, affirms that this manuscript is an honest, accurate, and transparent account of the study being reported; that no important aspects of the study have been omitted; and that any discrepancies from the study as planned (and, if relevant, registered) have been explained.

## Supporting information

Supplementary Table 1.

Supplementary Table 2.

## Data Availability

Data can be made available by the corresponding author upon reasonable request.
