# Supplementary material for: Unraveling the Genetic and Environmental Risk Factors of Autism Spectrum Disorder Through a Case‐Control Study in Armenia
Source: Health Sci Rep. 2025 May 4;8(5):e70801. doi: 10.1002/hsr2.70801 (PMC12050264; doi:10.1002/hsr2.70801)
Supplement: Supplementary file 1 — Supplementary Table 1. [file HSR2-8-e70801-s001.docx]

**Supplementary Table 1**. **The summary of the distribution of various genetic probes between case and control groups.**

| Chromosomal Regions | Genetic probes | Value | Control  (148) | Case  (149) | P-Value |
| --- | --- | --- | --- | --- | --- |
| 15q11-15q13 region | 214 - SNRPN-HB2-85 probe 21014-L29483 | 0 | 96.60 | 80.82 | 88.74 |
|  |  | 1 | 3.40 | 19.18 | 11.26 |
|  |  | ***P-Value*** |  |  | ***0.0000*** |
|  | 475 - SNRPN-HB2-85 probe 12720-L13795 | 0 | 100.00 | 95.89 | 97.95 |
|  |  | 1 | 0.00 | 3.42 | 1.71 |
|  |  | 2 | 0.00 | 0.68 | 0.34 |
|  |  | ***P-Value*** |  |  | ***0.0458*** |
|  | 256 - UBE3A probe 01317-L12925 | 0 | 100.00 | 98.63 | 99.32 |
|  |  | 1 | 0.00 | 1.37 | 0.68 |
|  |  | ***P-Value*** |  |  | ***0.1545*** |
|  | 244 - UBE3A probe 10886-L14677 | 0 | 100.00 | 98.63 | 99.32 |
|  |  | 1 | 0.00 | 1.37 | 0.68 |
|  |  | ***P-Value*** |  |  | ***0.1545*** |
|  | 142 - UBE3A probe 10883-L11553 | 0 | 100.00 | 100.00 | 100.00 |
|  |  | ***P-Value*** |  |  | ***1.0000*** |
|  | 160 - UBE3A probe 04620-L14668 | 0 | 100.00 | 100.00 | 100.00 |
|  |  | ***P-Value*** |  |  | ***1.0000*** |
|  | 197 - UBE3A probe 10880-L11550 | 0 | 100.00 | 99.32 | 99.66 |
|  |  | 1 | 0.00 | 0.68 | 0.34 |
|  |  | ***P-Value*** |  |  | ***0.3148*** |
|  | 270 - ATP10A probe 11165-L12883 | 0 | 100.00 | 99.32 | 99.66 |
|  |  | 1 | 0.00 | 0.68 | 0.34 |
|  |  | ***P-Value*** |  |  | ***0.3148*** |
|  | 136 - ATP10A probe 12964-L14669 | 0 | 25.17 | 3.42 | 14.33 |
|  |  | 1 | 63.95 | 35.62 | 49.83 |
|  |  | 2 | 10.88 | 60.96 | 35.84 |
|  |  | ***P-Value*** |  |  | ***0.0000*** |
|  | 220 - GABRB3 probe 01315-L09339 | 0 | 99.32 | 99.32 | 99.32 |
|  |  | 1 | 0.68 | 0.68 | 0.68 |
|  |  | ***P-Value*** |  |  | ***0.9961*** |
|  | 292 - GABRB3 probe 10875-L11545 | 0 | 100.00 | 100.00 | 100.00 |
|  |  | ***P-Value*** |  |  | ***1.0000*** |
|  | 382 - GABRB3 probe 10874-L11544 | 0 | 100.00 | 97.95 | 98.98 |
|  |  | 1 | 0.00 | 2.05 | 1.02 |
|  |  | ***P-Value*** |  |  | ***0.0806*** |
|  | 148 - GABRB3 probe 10872-L11542 | 0 | 100.00 | 100.00 | 100.00 |
|  |  | ***P-Value*** |  |  | ***1.0000*** |
|  | 319 - GABRB3 probe 10870-L11540 | 0 | 100.00 | 99.32 | 99.66 |
|  |  | 1 | 0.00 | 0.68 | 0.34 |
|  |  | ***P-Value*** |  |  | ***0.3148*** |
|  | 184 - GABRB3 probe 10868-L11538 | 0 | 100.00 | 100.00 | 100.00 |
|  |  | ***P-Value*** |  |  | ***1.0000*** |
|  | 355 - GABRB3 probe 10867-L11537 | 0 | 14.29 | 3.42 | 8.87 |
|  |  | 1 | 80.27 | 30.82 | 55.63 |
|  |  | 2 | 5.44 | 65.75 | 35.49 |
|  |  | ***P-Value*** |  |  | ***0.0000*** |
|  | 436 - OCA2 probe 02040-L01553 | 0 | 99.32 | 70.55 | 84.98 |
|  |  | 1 | 0.68 | 29.45 | 15.02 |
|  |  | ***P-Value*** |  |  | ***0.0000*** |
|  | 445 - OCA2 probe 02041-L03725 | 0 | 31.97 | 2.05 | 17.06 |
|  |  | 1 | 65.99 | 46.58 | 56.31 |
|  |  | 2 | 2.04 | 51.37 | 26.62 |
|  |  | ***P-Value*** |  |  | ***0.0000*** |
|  | 492 - SCG5 probe 12954-L14464 | 0 | 100.00 | 100.00 | 100.00 |
|  |  | ***P-Value*** |  |  | ***1.0000*** |
|  | 427 - SCG5 probe 12951-L29660 | 0 | 100.00 | 99.32 | 99.66 |
|  |  | 1 | 0.00 | 0.68 | 0.34 |
|  |  | ***P-Value*** |  |  | ***0.3148*** |
|  | 202 - APBA2 probe 01314-L00867 | 0 | 71.43 | 19.86 | 45.73 |
|  |  | 1 | 28.57 | 73.97 | 51.19 |
|  |  | 2 | 0.00 | 6.16 | 3.07 |
|  |  | ***P-Value*** |  |  | ***0.0000*** |
|  | 178 - NDNL2 probe 08377-L08231 | 0 | 97.28 | 57.53 | 77.47 |
|  |  | 1 | 2.72 | 42.47 | 22.53 |
|  |  | ***P-Value*** |  |  | ***0.0000*** |
|  | 300 - TJP1 probe 08389-L14671 | 0 | 47.62 | 0.00 | 23.89 |
|  |  | 1 | 51.70 | 75.34 | 63.48 |
|  |  | 2 | 0.68 | 24.66 | 12.63 |
|  |  | ***P-Value*** |  |  | ***0.0000*** |
|  | 373 - TRPM1 probe 08397-L14672 | 0 | 100.00 | 100.00 | 100.00 |
|  |  | ***P-Value*** |  |  | ***1.0000*** |
|  | 166 - KLF13 probe 08376-L08230 | 0 | 27.21 | 13.01 | 20.14 |
|  |  | 1 | 70.75 | 65.75 | 68.26 |
|  |  | 2 | 2.04 | 21.23 | 11.60 |
|  |  | ***P-Value*** |  |  | ***0.0000*** |
|  | 286 - CHRNA7 probe 12956-L08237 | 0 | 100.00 | 96.58 | 98.29 |
|  |  | 1 | 0.00 | 3.42 | 1.71 |
|  |  | ***P-Value*** |  |  | ***0.0236*** |
| 16p11 region | 483 - LAT probe 11677-L12448 | 0 | 95.92 | 17.12 | 56.66 |
|  |  | 1 | 4.08 | 82.88 | 43.34 |
|  |  | ***P-Value*** |  |  | ***0.0000*** |
|  | 364 - SPN probe 11672-L12443 | 0 | 93.88 | 43.84 | 68.94 |
|  |  | 1 | 6.12 | 56.16 | 31.06 |
|  |  | ***P-Value*** |  |  | ***0.0000*** |
|  | 238 - MAZ probe 11669-L12440 | 0 | 76.19 | 13.70 | 45.05 |
|  |  | 1 | 23.81 | 76.03 | 49.83 |
|  |  | 2 | 0.00 | 10.27 | 5.12 |
|  |  | ***P-Value*** |  |  | ***0.0000*** |
|  | 420 - MAZ probe 11673-L29557 | 0 | 61.22 | 13.70 | 37.54 |
|  |  | 1 | 38.78 | 80.14 | 59.39 |
|  |  | 2 | 0.00 | 6.16 | 3.07 |
|  |  | ***P-Value*** |  |  | ***0.0000*** |
|  | 346 - MVP probe 00550-L22423 | 0 | 97.96 | 97.26 | 97.61 |
|  |  | 1 | 2.04 | 2.74 | 2.39 |
|  |  | ***P-Value*** |  |  | ***0.6953*** |
|  | 208 - SEZ6L2 probe 11668-L12439 | 0 | 82.99 | 19.86 | 51.54 |
|  |  | 1 | 17.01 | 77.40 | 47.10 |
|  |  | 2 | 0.00 | 2.74 | 1.37 |
|  |  | ***P-Value*** |  |  | ***0.0000*** |
|  | 454 - HIRIP3 probe 11674-L12445 | 0 | 75.51 | 13.01 | 44.37 |
|  |  | 1 | 24.49 | 83.56 | 53.92 |
|  |  | 2 | 0.00 | 3.42 | 1.71 |
|  |  | ***P-Value*** |  |  | ***0.0000*** |
|  | 172 - HIRIP3 probe 11667-L14670 | 0 | 57.14 | 32.19 | 44.71 |
|  |  | 1 | 42.18 | 67.81 | 54.95 |
|  |  | 2 | 0.68 | 0.00 | 0.34 |
|  |  | ***P-Value*** |  |  | ***0.0000*** |
|  | 226 - DOC2A probe 13162-L12447 | 0 | 78.23 | 23.97 | 51.19 |
|  |  | 1 | 21.77 | 73.97 | 47.78 |
|  |  | 2 | 0.00 | 2.05 | 1.02 |
|  |  | ***P-Value*** |  |  | ***0.0000*** |
|  | 465 - MAPK3 probe 11675-L12446 | 0 | 87.07 | 18.49 | 52.90 |
|  |  | 1 | 12.93 | 81.51 | 47.10 |
|  |  | ***P-Value*** |  |  | ***0.0000*** |
|  | 337 - CD2BP2 probe 11671-L12442 | 0 | 99.32 | 32.88 | 66.21 |
|  |  | 1 | 0.68 | 67.12 | 33.79 |
|  |  | ***P-Value*** |  |  | ***0.0000*** |
|  | 310 - SHANK3 probe 20567-L14007 | 0 | 57.82 | 12.33 | 35.15 |
|  |  | 1 | 42.18 | 82.19 | 62.12 |
|  |  | 2 | 0.00 | 5.48 | 2.73 |
|  |  | ***P-Value*** |  |  | ***0.0000*** |
|  | 391 - SHANK3 probe 14190-L15800 | 0 | 89.80 | 17.81 | 53.92 |
|  |  | 1 | 10.20 | 74.66 | 42.32 |
|  |  | 2 | 0.00 | 7.53 | 3.75 |
|  |  | ***P-Value*** |  |  | ***0.0000*** |
|  | 232 - SHANK3 probe 06787-L07383 | 0 | 28.57 | 3.42 | 16.04 |
|  |  | 1 | 69.39 | 58.90 | 64.16 |
|  |  | 2 | 2.04 | 37.67 | 19.80 |
|  |  | ***P-Value*** |  |  | ***0.0000*** |
| 11q13 region | 160 - SHANK2 probe 16545-L19036 | 0 | 100.00 | 97.95 | 98.98 |
|  |  | 1 | 0.00 | 2.05 | 1.02 |
|  |  | ***P-Value*** |  |  | ***0.0806*** |
|  | 327 - SHANK2 probe 16561-L19764 | 0 | 96.60 | 86.30 | 91.47 |
|  |  | 1 | 3.40 | 13.70 | 8.53 |
|  |  | ***P-Value*** |  |  | ***0.0016*** |
|  | 408 - SHANK2 probe 16568-L19059 | 0 | 100.00 | 86.30 | 93.17 |
|  |  | 1 | 0.00 | 13.70 | 6.83 |
|  |  | ***P-Value*** |  |  | ***0.0000*** |
|  | 288 - SHANK2 probe 16558-SP0376-L19763 | 0 | 98.64 | 58.22 | 78.50 |
|  |  | 1 | 1.36 | 33.56 | 17.41 |
|  |  | 2 | 0.00 | 8.22 | 4.10 |
|  |  | ***P-Value*** |  |  | ***0.0000*** |
|  | 142 - SHANK2 probe 16543-L19034 | 0 | 65.99 | 59.59 | 62.80 |
|  |  | 1 | 34.01 | 40.41 | 37.20 |
|  |  | ***P-Value*** |  |  | ***0.2573*** |
|  | 211 - SHANK2 probe 16551-L19042 | 0 | 98.64 | 93.84 | 96.25 |
|  |  | 1 | 1.36 | 6.16 | 3.75 |
|  |  | ***P-Value*** |  |  | ***0.0305*** |
|  | 364 - SHANK2 probe 16564-L19055 | 0 | 93.88 | 65.75 | 79.86 |
|  |  | 1 | 6.12 | 33.56 | 19.80 |
|  |  | 2 | 0.00 | 0.68 | 0.34 |
|  |  | ***P-Value*** |  |  | ***0.0000*** |
|  | 265 - SHANK2 probe 16556-L19047 | 0 | 98.64 | 74.66 | 86.69 |
|  |  | 1 | 1.36 | 23.97 | 12.63 |
|  |  | 2 | 0.00 | 1.37 | 0.68 |
|  |  | ***P-Value*** |  |  | ***0.0000*** |
|  | 436 - SHANK2 probe 16570-L19765 | 0 | 61.22 | 31.51 | 46.42 |
|  |  | 1 | 38.78 | 63.70 | 51.19 |
|  |  | 2 | 0.00 | 4.79 | 2.39 |
|  |  | ***P-Value*** |  |  | ***0.0000*** |
|  | 191 - SHANK2 probe 16549-L19760 | 0 | 90.48 | 67.81 | 79.18 |
|  |  | 1 | 9.52 | 32.19 | 20.82 |
|  |  | ***P-Value*** |  |  | ***0.0000*** |
|  | 391 - SHANK2 probe 16566-L19057 | 0 | 88.44 | 93.84 | 91.13 |
|  |  | 1 | 11.56 | 6.16 | 8.87 |
|  |  | ***P-Value*** |  |  | ***0.1041*** |
|  | 355 - SHANK2 probe 16563-L19054 | 0 | 98.64 | 93.84 | 96.25 |
|  |  | 1 | 1.36 | 6.16 | 3.75 |
|  |  | ***P-Value*** |  |  | ***0.0305*** |
|  | 185 - SHANK2 probe 16548-L19759 | 0 | 86.39 | 34.93 | 60.75 |
|  |  | 1 | 13.61 | 45.89 | 29.69 |
|  |  | 2 | 0.00 | 19.18 | 9.56 |
|  |  | ***P-Value*** |  |  | ***0.0000*** |
|  | 136 - SHANK2 probe 16542-L19033 | 0 | 62.59 | 21.23 | 41.98 |
|  |  | 1 | 37.41 | 59.59 | 48.46 |
|  |  | 2 | 0.00 | 19.18 | 9.56 |
|  |  | ***P-Value*** |  |  | ***0.0000*** |
|  | 301 - SHANK2 probe 16559-L19050 | 0 | 86.39 | 88.36 | 87.37 |
|  |  | 1 | 13.61 | 11.64 | 12.63 |
|  |  | ***P-Value*** |  |  | ***0.6133*** |
|  | 254 - SHANK2 probe 16555-L19762 | 0 | 89.80 | 58.90 | 74.40 |
|  |  | 1 | 10.20 | 40.41 | 25.26 |
|  |  | 2 | 0.00 | 0.68 | 0.34 |
|  |  | ***P-Value*** |  |  | ***0.0000*** |
|  | 337 - SHANK2 probe 16562-L19053 | 0 | 100.00 | 97.26 | 98.63 |
|  |  | 1 | 0.00 | 2.74 | 1.37 |
|  |  | ***P-Value*** |  |  | ***0.0433*** |
|  | 148 - SHANK2 probe 16544-L19035 | 0 | 98.64 | 97.26 | 97.95 |
|  |  | 1 | 1.36 | 2.74 | 2.05 |
|  |  | ***P-Value*** |  |  | ***0.4046*** |
|  | 319 - SHANK2 probe 16560-L19051 | 0 | 74.83 | 47.95 | 61.43 |
|  |  | 1 | 25.17 | 50.68 | 37.88 |
|  |  | 2 | 0.00 | 1.37 | 0.68 |
|  |  | ***P-Value*** |  |  | ***0.0000*** |
|  | 283 - SHANK2 probe 16557-L19048 | 0 | 97.96 | 62.33 | 80.20 |
|  |  | 1 | 2.04 | 35.62 | 18.77 |
|  |  | 2 | 0.00 | 2.05 | 1.02 |
|  |  | ***P-Value*** |  |  | ***0.0000*** |
|  | 166 - SHANK2 probe 16546-L19037 | 0 | 61.90 | 45.21 | 53.58 |
|  |  | 1 | 38.10 | 54.79 | 46.42 |
|  |  | ***P-Value*** |  |  | ***0.0042*** |
|  | 229 - SHANK2 probe 16553-L19044 | 0 | 74.83 | 25.34 | 50.17 |
|  |  | 1 | 25.17 | 64.38 | 44.71 |
|  |  | 2 | 0.00 | 10.27 | 5.12 |
|  |  | ***P-Value*** |  |  | ***0.0000*** |
|  | 199 - SHANK2 probe 16550-SP0375-L19761 | 0 | 100.00 | 59.59 | 79.86 |
|  |  | 1 | 0.00 | 36.30 | 18.09 |
|  |  | 2 | 0.00 | 4.11 | 2.05 |
|  |  | ***P-Value*** |  |  | ***0.0000*** |
|  | 172 - SHANK2 probe 16547-L19038 | 0 | 76.19 | 60.27 | 68.26 |
|  |  | 1 | 23.81 | 36.99 | 30.38 |
|  |  | 2 | 0.00 | 2.74 | 1.37 |
|  |  | ***P-Value*** |  |  | ***0.0042*** |
|  | 247 - SHANK2 probe 16554-L19045 | 0 | 99.32 | 89.04 | 94.20 |
|  |  | 1 | 0.68 | 10.96 | 5.80 |
|  |  | ***P-Value*** |  |  | ***0.0002*** |
|  | 445 - SHANK2 probe 16571-L19062 | 0 | 66.67 | 19.86 | 43.34 |
|  |  | 1 | 33.33 | 74.66 | 53.92 |
|  |  | 2 | 0.00 | 5.48 | 2.73 |
|  |  | ***P-Value*** |  |  | ***0.0000*** |
|  | 427 - SHANK2 probe 16569-L19060 | 0 | 59.86 | 44.52 | 52.22 |
|  |  | 1 | 40.14 | 55.48 | 47.78 |
|  |  | ***P-Value*** |  |  | ***0.0086*** |
|  | 220 - SHANK2 probe 16552-L19043 | 0 | 89.12 | 53.42 | 71.33 |
|  |  | 1 | 10.88 | 46.58 | 28.67 |
|  |  | ***P-Value*** |  |  | ***0.0000*** |
|  | 400 - SHANK2 probe 16567-L19058 | 0 | 55.10 | 43.15 | 49.15 |
|  |  | 1 | 44.90 | 56.85 | 50.85 |
|  |  | ***P-Value*** |  |  | ***0.0408*** |
|  | 373 - SHANK2 probe 16565-L19056 | 0 | 91.84 | 71.23 | 81.57 |
|  |  | 1 | 8.16 | 28.77 | 18.43 |
|  |  | ***P-Value*** |  |  | ***0.0000*** |
|  | 0-no mutation, 1-heterozygous mutation, 2-homozygous mutation;  p<.05 was considered statistically significant for this study | | | | |
