# Supplementary material for: Unraveling the Genetic and Environmental Risk Factors of Autism Spectrum Disorder Through a Case‐Control Study in Armenia
Source: Health Sci Rep. 2025 May 4;8(5):e70801. doi: 10.1002/hsr2.70801 (PMC12050264; doi:10.1002/hsr2.70801)
Supplement: Supplementary file 2 — Supplementary Table 2. [file HSR2-8-e70801-s002.docx]

**Supplementary Table 2. The summary of Genetic Mutations Distribution by Gender.** Statistically significant differences, marked with an asterisk (*), highlight notable gender disparities within the case group.

|  |  | Case | | | | Control | | | |
| --- | --- | --- | --- | --- | --- | --- | --- | --- | --- |
|  |  | **Male** | | **Female** | | **Male** | | **Female** | |
| Genetic probes | Value | Count | Column N % | Count | Column N % | Count | Column N % | Count | Column N % |
| 214 - SNRPN-HB2-85 probe 21014-L29483* | 0 | 101 | 84.20% | 17 | 65.40% | 71 | 98.60% | 71 | 94.70% |
|  | 1 | 19 | 15.80% | 9 | 34.60% | 1 | 1.40% | 4 | 5.30% |
| 475 - SNRPN-HB2-85 probe 12720-L13795 | 0 | 114 | 95.00% | 26 | 100.00% | 72 | 100.00% | 75 | 100.00% |
|  | 1 | 5 | 4.20% | 0 | 0.00% | 0 | 0.00% | 0 | 0.00% |
|  | 2 | 1 | 0.80% | 0 | 0.00% | 0 | 0.00% | 0 | 0.00% |
| 256 - UBE3A probe 01317-L12925 | 0 | 118 | 98.30% | 26 | 100.00% | 72 | 100.00% | 75 | 100.00% |
|  | 1 | 2 | 1.70% | 0 | 0.00% | 0 | 0.00% | 0 | 0.00% |
| 244 - UBE3A probe 10886-L14677 | 0 | 119 | 99.20% | 25 | 96.20% | 72 | 100.00% | 75 | 100.00% |
|  | 1 | 1 | 0.80% | 1 | 3.80% | 0 | 0.00% | 0 | 0.00% |
| 142 - UBE3A probe 10883-L11553 | 0 | 120 | 100.00% | 26 | 100.00% | 72 | 100.00% | 75 | 100.00% |
| 160 - UBE3A probe 04620-L14668 | 0 | 120 | 100.00% | 26 | 100.00% | 72 | 100.00% | 75 | 100.00% |
| 197 - UBE3A probe 10880-L11550 | 0 | 119 | 99.20% | 26 | 100.00% | 72 | 100.00% | 75 | 100.00% |
|  | 1 | 1 | 0.80% | 0 | 0.00% | 0 | 0.00% | 0 | 0.00% |
| 270 - ATP10A probe 11165-L12883 | 0 | 119 | 99.20% | 26 | 100.00% | 72 | 100.00% | 75 | 100.00% |
|  | 1 | 1 | 0.80% | 0 | 0.00% | 0 | 0.00% | 0 | 0.00% |
| 136 - ATP10A probe 12964-L14669 | 0 | 5 | 4.20% | 0 | 0.00% | 15 | 20.80% | 22 | 29.30% |
|  | 1 | 45 | 37.50% | 7 | 26.90% | 49 | 68.10% | 45 | 60.00% |
|  | 2 | 70 | 58.30% | 19 | 73.10% | 8 | 11.10% | 8 | 10.70% |
| 220 - GABRB3 probe 01315-L09339 | 0 | 119 | 99.20% | 26 | 100.00% | 72 | 100.00% | 74 | 98.70% |
|  | 1 | 1 | 0.80% | 0 | 0.00% | 0 | 0.00% | 1 | 1.30% |
| 292 - GABRB3 probe 10875-L11545 | 0 | 120 | 100.00% | 26 | 100.00% | 72 | 100.00% | 75 | 100.00% |
| 382 - GABRB3 probe 10874-L11544 | 0 | 117 | 97.50% | 26 | 100.00% | 72 | 100.00% | 75 | 100.00% |
|  | 1 | 3 | 2.50% | 0 | 0.00% | 0 | 0.00% | 0 | 0.00% |
| 148 - GABRB3 probe 10872-L11542 | 0 | 120 | 100.00% | 26 | 100.00% | 72 | 100.00% | 75 | 100.00% |
| 319 - GABRB3 probe 10870-L11540 | 0 | 119 | 99.20% | 26 | 100.00% | 72 | 100.00% | 75 | 100.00% |
|  | 1 | 1 | 0.80% | 0 | 0.00% | 0 | 0.00% | 0 | 0.00% |
| 184 - GABRB3 probe 10868-L11538 | 0 | 120 | 100.00% | 26 | 100.00% | 72 | 100.00% | 75 | 100.00% |
| 355 - GABRB3 probe 10867-L11537 | 0 | 5 | 4.20% | 0 | 0.00% | 16 | 22.20% | 5 | 6.70% |
|  | 1 | 38 | 31.70% | 7 | 26.90% | 51 | 70.80% | 67 | 89.30% |
|  | 2 | 77 | 64.20% | 19 | 73.10% | 5 | 6.90% | 3 | 4.00% |
| 436 - OCA2 probe 02040-L01553 | 0 | 86 | 71.70% | 17 | 65.40% | 71 | 98.60% | 75 | 100.00% |
|  | 1 | 34 | 28.30% | 9 | 34.60% | 1 | 1.40% | 0 | 0.00% |
| 445 - OCA2 probe 02041-L03725 | 0 | 3 | 2.50% | 0 | 0.00% | 20 | 27.80% | 27 | 36.00% |
|  | 1 | 59 | 49.20% | 9 | 34.60% | 50 | 69.40% | 47 | 62.70% |
|  | 2 | 58 | 48.30% | 17 | 65.40% | 2 | 2.80% | 1 | 1.30% |
| 492 - SCG5 probe 12954-L14464 | 0 | 120 | 100.00% | 26 | 100.00% | 72 | 100.00% | 75 | 100.00% |
| 427 - SCG5 probe 12951-L29660 | 0 | 119 | 99.20% | 26 | 100.00% | 72 | 100.00% | 75 | 100.00% |
|  | 1 | 1 | 0.80% | 0 | 0.00% | 0 | 0.00% | 0 | 0.00% |
| 202 - APBA2 probe 01314-L00867* | 0 | 27 | 22.50% | 2 | 7.70% | 51 | 70.80% | 54 | 72.00% |
|  | 1 | 88 | 73.30% | 20 | 76.90% | 21 | 29.20% | 21 | 28.00% |
|  | 2 | 5 | 4.20% | 4 | 15.40% | 0 | 0.00% | 0 | 0.00% |
| 178 - NDNL2 probe 08377-L08231 | 0 | 68 | 56.70% | 16 | 61.50% | 68 | 94.40% | 75 | 100.00% |
|  | 1 | 52 | 43.30% | 10 | 38.50% | 4 | 5.60% | 0 | 0.00% |
| 300 - TJP1 probe 08389-L14671 | 0 | 0 | 0.00% | 0 | 0.00% | 32 | 44.40% | 38 | 50.70% |
|  | 1 | 88 | 73.30% | 22 | 84.60% | 40 | 55.60% | 36 | 48.00% |
|  | 2 | 32 | 26.70% | 4 | 15.40% | 0 | 0.00% | 1 | 1.30% |
| 373 - TRPM1 probe 08397-L14672 | 0 | 120 | 100.00% | 26 | 100.00% | 72 | 100.00% | 75 | 100.00% |
| 166 - KLF13 probe 08376-L08230 | 0 | 18 | 15.00% | 1 | 3.80% | 20 | 27.80% | 20 | 26.70% |
|  | 1 | 77 | 64.20% | 19 | 73.10% | 51 | 70.80% | 53 | 70.70% |
|  | 2 | 25 | 20.80% | 6 | 23.10% | 1 | 1.40% | 2 | 2.70% |
| 286 - CHRNA7 probe 12956-L08237 | 0 | 115 | 95.80% | 26 | 100.00% | 72 | 100.00% | 75 | 100.00% |
|  | 1 | 5 | 4.20% | 0 | 0.00% | 0 | 0.00% | 0 | 0.00% |
| 483 - LAT probe 11677-L12448* | 0 | 24 | 20.00% | 1 | 3.80% | 68 | 94.40% | 73 | 97.30% |
|  | 1 | 96 | 80.00% | 25 | 96.20% | 4 | 5.60% | 2 | 2.70% |
| 364 - SPN probe 11672-L12443* | 0 | 60 | 50.00% | 4 | 15.40% | 67 | 93.10% | 71 | 94.70% |
|  | 1 | 60 | 50.00% | 22 | 84.60% | 5 | 6.90% | 4 | 5.30% |
| 238 - MAZ probe 11669-L12440 | 0 | 18 | 15.00% | 2 | 7.70% | 51 | 70.80% | 61 | 81.30% |
|  | 1 | 90 | 75.00% | 21 | 80.80% | 21 | 29.20% | 14 | 18.70% |
|  | 2 | 12 | 10.00% | 3 | 11.50% | 0 | 0.00% | 0 | 0.00% |
| 420 - MAZ probe 11673-L29557 | 0 | 17 | 14.20% | 3 | 11.50% | 43 | 59.70% | 47 | 62.70% |
|  | 1 | 96 | 80.00% | 21 | 80.80% | 29 | 40.30% | 28 | 37.30% |
|  | 2 | 7 | 5.80% | 2 | 7.70% | 0 | 0.00% | 0 | 0.00% |
| 346 - MVP probe 00550-L22423 | 0 | 116 | 96.70% | 26 | 100.00% | 71 | 98.60% | 73 | 97.30% |
|  | 1 | 4 | 3.30% | 0 | 0.00% | 1 | 1.40% | 2 | 2.70% |
| 208 - SEZ6L2 probe 11668-L12439 | 0 | 27 | 22.50% | 2 | 7.70% | 57 | 79.20% | 65 | 86.70% |
|  | 1 | 91 | 75.80% | 22 | 84.60% | 15 | 20.80% | 10 | 13.30% |
|  | 2 | 2 | 1.70% | 2 | 7.70% | 0 | 0.00% | 0 | 0.00% |
| 454 - HIRIP3 probe 11674-L12445 | 0 | 18 | 15.00% | 1 | 3.80% | 51 | 70.80% | 60 | 80.00% |
|  | 1 | 97 | 80.80% | 25 | 96.20% | 21 | 29.20% | 15 | 20.00% |
|  | 2 | 5 | 4.20% | 0 | 0.00% | 0 | 0.00% | 0 | 0.00% |
| 172 - HIRIP3 probe 11667-L14670* | 0 | 43 | 35.80% | 4 | 15.40% | 39 | 54.20% | 45 | 60.00% |
|  | 1 | 77 | 64.20% | 22 | 84.60% | 32 | 44.40% | 30 | 40.00% |
|  | 2 | 0 | 0.00% | 0 | 0.00% | 1 | 1.40% | 0 | 0.00% |
| 226 - DOC2A probe 13162-L12447 | 0 | 32 | 26.70% | 3 | 11.50% | 57 | 79.20% | 58 | 77.30% |
|  | 1 | 85 | 70.80% | 23 | 88.50% | 15 | 20.80% | 17 | 22.70% |
|  | 2 | 3 | 2.50% | 0 | 0.00% | 0 | 0.00% | 0 | 0.00% |
| 465 - MAPK3 probe 11675-L12446* | 0 | 26 | 21.70% | 1 | 3.80% | 63 | 87.50% | 65 | 86.70% |
|  | 1 | 94 | 78.30% | 25 | 96.20% | 9 | 12.50% | 10 | 13.30% |
| 337 - CD2BP2 probe 11671-L12442* | 0 | 45 | 37.50% | 3 | 11.50% | 71 | 98.60% | 75 | 100.00% |
|  | 1 | 75 | 62.50% | 23 | 88.50% | 1 | 1.40% | 0 | 0.00% |
| 310 - SHANK3 probe 20567-L14007 | 0 | 17 | 14.20% | 1 | 3.80% | 43 | 59.70% | 42 | 56.00% |
|  | 1 | 96 | 80.00% | 24 | 92.30% | 29 | 40.30% | 33 | 44.00% |
|  | 2 | 7 | 5.80% | 1 | 3.80% | 0 | 0.00% | 0 | 0.00% |
| 391 - SHANK3 probe 14190-L15800 | 0 | 23 | 19.20% | 3 | 11.50% | 64 | 88.90% | 68 | 90.70% |
|  | 1 | 90 | 75.00% | 19 | 73.10% | 8 | 11.10% | 7 | 9.30% |
|  | 2 | 7 | 5.80% | 4 | 15.40% | 0 | 0.00% | 0 | 0.00% |
| 232 - SHANK3 probe 06787-L07383 | 0 | 5 | 4.20% | 0 | 0.00% | 19 | 26.40% | 23 | 30.70% |
|  | 1 | 73 | 60.80% | 13 | 50.00% | 50 | 69.40% | 52 | 69.30% |
|  | 2 | 42 | 35.00% | 13 | 50.00% | 3 | 4.20% | 0 | 0.00% |
| 160 - SHANK2 probe 16545-L19036 | 0 | 117 | 97.50% | 26 | 100.00% | 72 | 100.00% | 75 | 100.00% |
|  | 1 | 3 | 2.50% | 0 | 0.00% | 0 | 0.00% | 0 | 0.00% |
| 327 - SHANK2 probe 16561-L19764 | 0 | 102 | 85.00% | 24 | 92.30% | 69 | 95.80% | 73 | 97.30% |
|  | 1 | 18 | 15.00% | 2 | 7.70% | 3 | 4.20% | 2 | 2.70% |
| 408 - SHANK2 probe 16568-L19059 | 0 | 106 | 88.30% | 20 | 76.90% | 72 | 100.00% | 75 | 100.00% |
|  | 1 | 14 | 11.70% | 6 | 23.10% | 0 | 0.00% | 0 | 0.00% |
| 288 - SHANK2 probe 16558-SP0376-L19763 | 0 | 70 | 58.30% | 15 | 57.70% | 71 | 98.60% | 74 | 98.70% |
|  | 1 | 42 | 35.00% | 7 | 26.90% | 1 | 1.40% | 1 | 1.30% |
|  | 2 | 8 | 6.70% | 4 | 15.40% | 0 | 0.00% | 0 | 0.00% |
| 142 - SHANK2 probe 16543-L19034 | 0 | 75 | 62.50% | 12 | 46.20% | 49 | 68.10% | 48 | 64.00% |
|  | 1 | 45 | 37.50% | 14 | 53.80% | 23 | 31.90% | 27 | 36.00% |
| 211 - SHANK2 probe 16551-L19042 | 0 | 113 | 94.20% | 24 | 92.30% | 71 | 98.60% | 74 | 98.70% |
|  | 1 | 7 | 5.80% | 2 | 7.70% | 1 | 1.40% | 1 | 1.30% |
| 364 - SHANK2 probe 16564-L19055 | 0 | 80 | 66.70% | 16 | 61.50% | 65 | 90.30% | 73 | 97.30% |
|  | 1 | 39 | 32.50% | 10 | 38.50% | 7 | 9.70% | 2 | 2.70% |
|  | 2 | 1 | 0.80% | 0 | 0.00% | 0 | 0.00% | 0 | 0.00% |
| 265 - SHANK2 probe 16556-L19047 | 0 | 90 | 75.00% | 19 | 73.10% | 71 | 98.60% | 74 | 98.70% |
|  | 1 | 29 | 24.20% | 6 | 23.10% | 1 | 1.40% | 1 | 1.30% |
|  | 2 | 1 | 0.80% | 1 | 3.80% | 0 | 0.00% | 0 | 0.00% |
| 436 - SHANK2 probe 16570-L19765 | 0 | 38 | 31.70% | 8 | 30.80% | 47 | 65.30% | 43 | 57.30% |
|  | 1 | 77 | 64.20% | 16 | 61.50% | 25 | 34.70% | 32 | 42.70% |
|  | 2 | 5 | 4.20% | 2 | 7.70% | 0 | 0.00% | 0 | 0.00% |
| 191 - SHANK2 probe 16549-L19760* | 0 | 80 | 66.70% | 19 | 73.10% | 61 | 84.70% | 72 | 96.00% |
|  | 1 | 40 | 33.30% | 7 | 26.90% | 11 | 15.30% | 3 | 4.00% |
| 391 - SHANK2 probe 16566-L19057 | 0 | 111 | 92.50% | 26 | 100.00% | 61 | 84.70% | 69 | 92.00% |
|  | 1 | 9 | 7.50% | 0 | 0.00% | 11 | 15.30% | 6 | 8.00% |
| 355 - SHANK2 probe 16563-L19054 | 0 | 112 | 93.30% | 25 | 96.20% | 72 | 100.00% | 73 | 97.30% |
|  | 1 | 8 | 6.70% | 1 | 3.80% | 0 | 0.00% | 2 | 2.70% |
| 185 - SHANK2 probe 16548-L19759 | 0 | 42 | 35.00% | 9 | 34.60% | 60 | 83.30% | 67 | 89.30% |
|  | 1 | 54 | 45.00% | 13 | 50.00% | 12 | 16.70% | 8 | 10.70% |
|  | 2 | 24 | 20.00% | 4 | 15.40% | 0 | 0.00% | 0 | 0.00% |
| 136 - SHANK2 probe 16542-L19033 | 0 | 26 | 21.70% | 5 | 19.20% | 40 | 55.60% | 52 | 69.30% |
|  | 1 | 72 | 60.00% | 15 | 57.70% | 32 | 44.40% | 23 | 30.70% |
|  | 2 | 22 | 18.30% | 6 | 23.10% | 0 | 0.00% | 0 | 0.00% |
| 301 - SHANK2 probe 16559-L19050 | 0 | 104 | 86.70% | 25 | 96.20% | 61 | 84.70% | 66 | 88.00% |
|  | 1 | 16 | 13.30% | 1 | 3.80% | 11 | 15.30% | 9 | 12.00% |
| 254 - SHANK2 probe 16555-L19762 | 0 | 72 | 60.00% | 14 | 53.80% | 65 | 90.30% | 67 | 89.30% |
|  | 1 | 48 | 40.00% | 11 | 42.30% | 7 | 9.70% | 8 | 10.70% |
|  | 2 | 0 | 0.00% | 1 | 3.80% | 0 | 0.00% | 0 | 0.00% |
| 337 - SHANK2 probe 16562-L19053 | 0 | 116 | 96.70% | 26 | 100.00% | 72 | 100.00% | 75 | 100.00% |
|  | 1 | 4 | 3.30% | 0 | 0.00% | 0 | 0.00% | 0 | 0.00% |
| 148 - SHANK2 probe 16544-L19035 | 0 | 116 | 96.70% | 26 | 100.00% | 72 | 100.00% | 73 | 97.30% |
|  | 1 | 4 | 3.30% | 0 | 0.00% | 0 | 0.00% | 2 | 2.70% |
| 319 - SHANK2 probe 16560-L19051 | 0 | 56 | 46.70% | 14 | 53.80% | 56 | 77.80% | 54 | 72.00% |
|  | 1 | 63 | 52.50% | 11 | 42.30% | 16 | 22.20% | 21 | 28.00% |
|  | 2 | 1 | 0.80% | 1 | 3.80% | 0 | 0.00% | 0 | 0.00% |
| 283 - SHANK2 probe 16557-L19048 | 0 | 77 | 64.20% | 14 | 53.80% | 71 | 98.60% | 73 | 97.30% |
|  | 1 | 41 | 34.20% | 11 | 42.30% | 1 | 1.40% | 2 | 2.70% |
|  | 2 | 2 | 1.70% | 1 | 3.80% | 0 | 0.00% | 0 | 0.00% |
| 166 - SHANK2 probe 16546-L19037 | 0 | 54 | 45.00% | 12 | 46.20% | 43 | 59.70% | 48 | 64.00% |
|  | 1 | 66 | 55.00% | 14 | 53.80% | 29 | 40.30% | 27 | 36.00% |
| 229 - SHANK2 probe 16553-L19044 | 0 | 30 | 25.00% | 7 | 26.90% | 53 | 73.60% | 57 | 76.00% |
|  | 1 | 78 | 65.00% | 16 | 61.50% | 19 | 26.40% | 18 | 24.00% |
|  | 2 | 12 | 10.00% | 3 | 11.50% | 0 | 0.00% | 0 | 0.00% |
| 199 - SHANK2 probe 16550-SP0375-L19761 | 0 | 74 | 61.70% | 13 | 50.00% | 72 | 100.00% | 75 | 100.00% |
|  | 1 | 41 | 34.20% | 12 | 46.20% | 0 | 0.00% | 0 | 0.00% |
|  | 2 | 5 | 4.20% | 1 | 3.80% | 0 | 0.00% | 0 | 0.00% |
| 172 - SHANK2 probe 16547-L19038 | 0 | 74 | 61.70% | 14 | 53.80% | 54 | 75.00% | 58 | 77.30% |
|  | 1 | 42 | 35.00% | 12 | 46.20% | 18 | 25.00% | 17 | 22.70% |
|  | 2 | 4 | 3.30% | 0 | 0.00% | 0 | 0.00% | 0 | 0.00% |
| *Statistically significant difference in case group; | | | | | | | | | |
| 0-no mutation, 1-heterozygous mutation, 2-homozygous mutation;  p<.05 was considered statistically significant for this study. | | | | | | | | | |
